# Platelet Toll-Like-Receptor-2 and -4 Mediate Different Immune-Related Responses to Bacterial Ligands

**DOI:** 10.1055/a-1827-7365

**Published:** 2022-07-11

**Authors:** Marius Niklaus, Philipp Klingler, Katja Weber, Angela Koessler, Sabine Kuhn, Markus Boeck, Anna Kobsar, Juergen Koessler

**Affiliations:** 1 Institute of Clinical Transfusion Medicine and Haemotherapy, University of Wuerzburg, Wuerzburg, Germany

**Keywords:** platelet physiology, receptors, cell-cell interactions, immunity

## Abstract

**Background** Like immune cells, platelets express toll-like receptors (TLRs) on their surface membrane. TLR2 and TLR4 are able to recognize bacterial antigens and have the potential to influence hemostatic functions and classical intracellular signaling pathways. This study investigated the role of TLR2 and TLR4 for immune-related functions in human platelets.

**Materials and Methods** Washed platelets and neutrophils were prepared from fresh human peripheral blood. Basal-, Pam3CSK4- (as TLR2 agonist) and Lipopolysaccharides (LPS; as TLR4 agonist) -induced CD62P expression, fibrinogen binding and TLR2 or TLR4 expression, intracellular reactive oxygen species (ROS) production in H _2_ DCFDA-loaded platelets and uptake of fluorescence-labeled TLR ligands, and fluorophore-conjugated fibrinogen were evaluated by flow cytometry. Analysis of platelet–neutrophil complexes was performed after coincubation of washed platelets and neutrophils in the presence and absence of TLR2 or TLR4 agonists on poly-L-lysine coated surfaces, followed by immunostaining and immunofluorescence imaging.

**Results** Pam3CSK4 rapidly and transiently increased TLR2 and TLR4 expression. Over the course of 30 minutes after activation with Pam3CSK4 and LPS, the expression of both receptors decreased. Pam3CSK4-stimulated intracellular ROS production and the uptake of TLR ligands or fibrinogen much stronger than LPS. Besides, TLR4 activation led to a significant increase of platelet–neutrophil contacts.

**Conclusion** Stimulation leads to rapid mobilization of TLR2 or TLR4 to the platelet surface, presumably followed by receptor internalization along with bound TLR ligands. After activation, platelet TLR2 and TLR4 mediate different immune-related reactions. In particular, TLR2 induces intracellular responses in platelets, whereas TLR4 initiates interactions with other immune cells such as neutrophils.

## Introduction

Platelets are anucleated cell fragments derived from megakaryocytes and well known as essential actors in the processes of hemostasis or thrombus formation. In addition, an involvement in inflammatory and immunological mechanisms are attributed to platelets. 1 They have a repertoire of stored chemokines and several molecules on their membrane surface, like CD62P, enabling interactions with immune cells. 2

Platelets also bear toll-like receptors (TLRs), allowing the recognition of pathogen- or damage-associated molecular patterns. TLR2 and TLR4 are two important representatives of these receptors mediating intracellular activation via the MyD88-dependent pathway. 3 TLR2, forming heterodimers with TLR1 or TLR6, can be stimulated by acylated lipoproteins like the synthetic triacylated molecule Pam3CSK4. Lipopolysaccharides (LPS) from gram-negative bacteria, for example, *Escherichia coli* , are ligands for TLR4, appearing as homodimer associated with the glycosyl-phosphatidylinositol–anchored protein CD14. 4 5 6

TLR are considered to be a substantial part of innate immunity and to be involved in various clinical conditions driven by thromboinflammation. Platelet TLR may play a role in bacterial and viral infections but also in sterile inflammatory responses, cardiovascular disease, or transfusion-related adverse events, as comprehensively reviewed by Hally et al. 7

Recently, we could show that both receptors are able to activate nuclear factor κ-B (NFκB)-mediated and classical signaling pathways in platelets, with a higher intensity for TLR2. In contrast to TLR2, stimulation of TLR4 resulted in decreased Akt/protein kinase B phosphorylation, conditioned by enhanced protein phosphatase 2A activity. Furthermore, TLR4-mediated signaling induced platelet adhesion and facilitated ristocetin-induced platelet agglutination. 8

For the identification of possible targets to interrupt overshooting TLR activation, crucial in the context of microbial infections, autoimmune disorders, or in transfusion reactions, 7 it is important to further elucidate TLR-induced mechanisms contributing to platelet–leukocyte interactions.

In this study, we focused on TLR2- and TLR4-induced mechanisms on binding of the specific ligands Pam3CSK4 and LPS (from *E. coli* ), as potentially relevant in bacterial infections. According to previous studies, 8 9 the agonists were used in the lowest concentrations inducing or supporting platelet activation. We investigated the course of TLR2 and TLR4 expressions to estimate the time-dependent stimulatory capacity of these two receptors. In addition, the time-dependent expression of platelet activation markers was determined, including CD62P, which is a central binding partner for the formation of neutrophil extracellular traps (NET) via the P-selectin glycoprotein ligand-1 (PSGL-1). 10 TLR2- and TLR4-mediated surface adhesion and generation of platelet–leukocyte aggregates were analyzed, as these processes are essential prerequisites to initiate immune responses at the site of inflammation or vessel wall injuries. 11 Furthermore, the investigations comprised TLR2- and TLR4-induced production of reactive oxygen species (ROS), presumably an essential option to support defense against microbial antigens and to influence signaling pathways in platelets or immune cells. 12 13

We can show that platelets have an effective repertoire to exert functional responses and interactions with immune cells on differential TLR2 and TLR4 stimulation, highlighting the role of platelet TLR2 and TLR4 in processes of innate immunity.

## Materials and Methods

### Materials

Pam3CSK4 (no. tlrl-pms), specific rat polyclonal blocking antibodies for human TLR2 (#pab-hstlr2), TLR4 (#pab-hstlr4) and the corresponding isotype control (#maba2-ctrl), biotinylated ultrapure LPS from *E. coli* serotype O111:B4 smooth strain (#tlrl-lpsbiot), and biotinylated Pam3CSK4 (#tlrl-bpms) were from InvivoGen (Toulouse, France). Ethylene glycol-bis(β-aminoethyl ether)-N,N,N',N'-tetra-acetic acid (EGTA), apyrase (#A2230), prostaglandin E1 (PGE1; #P5515), LPS from *E. coli* serotype O111:B4 smooth strain (#L3024), thrombin from human plasma (#T6884), goat serum (GS), fetal bovine serum, bovine serum albumin (BSA), human plasma fibrinogen (#F3879), 4-(2-hydroxyethyl)-1-piperazineethanesulfonic acid (HEPES), Tyrode's salt solution, poly-L-lysine, TRITC-conjugated goat antimouse antibody (#T5393), fluorescein isothiocyanate (FITC)-conjugated goat antimouse polyclonal antibody (#F2012), and Trypan blue solution (#T8154) were from Sigma-Aldrich Chemie GmbH (Muenchen, Germany). TRAP-6 reagent (#HB-5512-FG) was from Hart Biologicals Ltd (Hartlepool, United Kingdom). Mouse monoclonal anti-TLR2 (#16–9922–82) and anti-TLR4 antibodies (#16–9917–82), mouse immunoglobulin (Ig)-G2a (isotype; #16–4724–82), Alexa Flour 488-conjugated fibrinogen (#F13191), mouse monoclonal antihuman CD41a antibody (#14–0419–82) and cell permeant 2',7'-Dichlordihydrofluorescein-diacetat (H _2_ DCFDA; #D399) were from Thermo Fisher Scientific (Darmstadt, Germany). FITC-conjugated mouse anti-CD62P antibody (#SM1150F) and the corresponding isotype control (#SM10F) were from OriGene Biomedical GmbH (Burladingen, Germany). FITC-conjugated mouse monoclonal anti-fibrinogen antibody clone 9F9 (#5009-F100T) and the corresponding isotype control (#5108-F100T) were from STAGO GmbH (Duesseldorf Germany). APC-conjugated mouse antihuman CD154 antibody (#648887), APC-conjugated mouse IgG1 (#345818), FITC-conjugated mouse monoclonal anti-human CD11b antibody (#562793) and FITC-Streptavidin (#554060) were from BD Bioscience (Heidelberg, Germany). ProLong Diamond Antifade Mountant was from Invitrogen (Thermo Fisher Scientific, Eugene, United States)

### Blood Collection and Preparation of Washed Platelets

Our studies with human platelets and the consent procedure were approved by the local Ethics Committee of the University of Wuerzburg (approval number: 101/15). All participants provided their written informed consent. The study was performed according to our institutional guidelines and to the Declaration of Helsinki.

Peripheral venous blood (PB) from informed healthy voluntary donors was collected in polypropylene tubes containing 3.2% citrate buffer (106 mM trisodium citrate, Sarstedt, Nuembrecht, Germany). To prepare washed platelets (WP), 3-mM EGTA was added to PB prior to centrifugation to prevent platelet activation. Platelet-rich plasma (PRP) was obtained by centrifugation at 280 × g for 5 minutes. Then, 75-nM PGE1 was added to PRP, and platelets were pelleted at 430 × g for 7 minutes. The pelleted platelets were then washed once in chloride/glucose/sodium (CGS) buffer (120-mM sodium chloride, 12.9-mM trisodium citrate, 30-mM D-glucose, and pH = 6.5) and resuspended in Tyrode's salt solution to a final concentration of 3 × 10 ^8^ platelets/mL.

### Measurement of Toll-Like Receptor-2 and -4 Expressions

Overall, 40 µL of WP was stained with 5 µg/mL anti-TLR2, anti-TLR4 antibodies, or isotype control for 10 minutes at 37°C. After that, 1-mM CaCl _2_ was added to WP followed by stimulation with incubation buffer, 15 or 100 µg/mL Pam3CSK4 or 15 or 100 µg/mL LPS for 1, 2, 5, 10, and 30 minutes. Samples were stopped with 1% formaldehyde (final concentration), fixed for 10 minutes at room temperature (RT) and centrifuged for 1 minute at 14,000 × g. The pellet was resuspended in 100 µL of phosphate buffered saline (PBS)/BSA/glucose (Glc) (Dulbecco's PBS [Ca ^2+^ , Mg ^2+^ free], 5.5-mM D-Glucose, 0.5% BSA) and stained at RT in the dark for 30 minutes with 5 µg/mL of FITC-conjugated goat antimouse antibody. Finally, samples were diluted with 300 µL of PBS/BSA/Glc and analyzed by flow cytometry using a FACSCalibur flow cytometer from Becton Dickinson (Franklin Lakes, New Jersey, United States) with CELLQuest software, version 6.0. The platelet population was identified by its forward and side scatter distribution and 10,000 events were analyzed for mean fluorescence.

### Measurement of Fibrinogen Binding, CD62P Expression, and CD40L Expression

For determination of fibrinogen binding on the platelet surface, 100 µg/mL of human plasma fibrinogen and 10 µg/mL of Alexa Flour 488-conjugated fibrinogen were added to 40 µL of WP and then incubated for 10 minutes at 37°C in the dark. For analysis of CD62P expression, 40 µL of WP was preincubated with 10 µg/mL of FITC-conjugated anti-CD62P antibody or isotype control for 10 minutes at 37°C in the dark. For detection of CD40L expression, 50 µL of WP (3 × 10 ^8^ platelets/mL) or 50 µL of PRP was incubated with 2 µL of APC-conjugated anti-CD154 antibody or isotype control for 10 minutes at 37°C in the dark.

Then, 1-mM CaCl _2_ (final concentration) was added to WP and platelet suspensions were stimulated with buffer, 15 µg/mL of Pam3CSK4, 15 µg/mL of LPS, and 0.5 U/mL of thrombin (for WP) or 10 µM of TRAP-6 (for PRP) for 5 minutes at 37°C. Samples were stopped with 1% formaldehyde (final concentration), fixed for 10 minutes at RT, diluted with 300 µL of PBS/BSA/Glc, and analyzed by flow cytometry as described above.

### Detection of Intracellular Reactive Oxygen Species Production

Intracellular ROS production was measured as described. 14 15 For this purpose, PRP was supplemented with 75 nM of PGE1 and 0.3 of U/mL apyrase. After centrifugation at 430 × g for 7 minutes, platelets (3 × 10 ^8^ per mL) were loaded for 30 minutes at 37°C in the dark with 5 µM of H _2_ DCFDA in PBS/BSA/Glc, supplemented with 1-mM EDTA and 0.3 U/mL of apyrase. Platelets loaded with H _2_ DCFDA were then pelleted for 7 minutes at 430 × g and resuspended in HEPES Buffer (150-mM NaCl, 5-mM KCl, 1-mM MCl _2_ , 10-mM D-Glucose, 10-mM HEPES, and pH = 7.4) without EDTA, PGE1, and apyrase. Platelet count was adjusted to 3 × 10 ^8^ platelets/mL. Platelets were left quiescent for 10 minutes at 37°C in the dark. To induce intracellular ROS production, 100 µL of H _2_ DCFDA loaded platelets was supplemented with 1-mM CaCl _2_ and stimulated with 15 µg/mL of TLR2 or TLR4 agonists as described above, in combination with 5 µg/mL blocking anti-TLR2 and anti-TLR4 antibody or the corresponding isotype controls. Samples were diluted after 2 minutes with 1 mL of PBS/BSA/Glc and analyzed by flow cytometry immediately. The measurements with subsequent analysis of platelet ROS were performed in the same gate, chosen in forward scatter (FSC) versus sideward scatter (SSC) coordinates, ensuring the selection of cells of the same size. In addition, the same number of cells (10,000 events) were included in the calculations for mean fluorescence intensity (MFI) in each sample.

### Measurement of Uptake of Fibrinogen in Platelets

Platelet uptake experiments were performed similar to Banerjee et al. 16 Briefly, WP were resuspended in HEPES buffer to 10 ^8^ platelets/mL, followed by incubation for 10 minutes at 37°C. After that resting period, 100 µL of WP was supplemented with 1 mM of CaCl _2_ and 60 µg/mL of Alexa Fluor 488-conjugated fibrinogen and stimulated for 1 hour with TLR2 or TLR4 agonists as described above. This stimulation was performed with constant shaking for 1 hour at 37°C in the dark. Platelets were then fixed with the equal volume of cold 4% paraformaldehyde (PFA) in PBS for 20 minutes on ice. After fixation, samples were diluted with 160 µL of PBS/BSA/Glc. For detection of intracellular Alexa Fluor 488 fluorescence, extracellular fluorescence was quenched with 0.1% Trypan blue. Afterwards, platelets were analyzed by flow cytometry as described above.

### Measurement of Uptake of Toll-Like Receptor-2and -4 Ligands

The uptake experiments with TLR2 or TLR4 ligands were performed similar to Banerjee et al. 16 Briefly, WP were resuspended in HEPES buffer to a concentration of 10 ^8^ platelets/mL, followed by incubation at 37°C for 10 minutes. Then, 50 µL of WP was supplemented with 1 mM of CaCl _2_ followed by stimulation with 15 µg/mL of biotin-conjugated Pam3CSK4 or 15 µg/mL of biotin-conjugated LPS, together with 10 µg/mL of FITC-conjugated streptavidin under constant shaking for 30 minutes at 37°C in the dark. After that, platelets were fixed with the equal volume of cold 4% PFA in PBS for 20 minutes on ice. After fixation, samples were diluted with 260 µL of PBS/BSA/Glc. For detection of intracellular Alexa Fluor 488 fluorescence, extracellular fluorescence was quenched with 0.1% Trypan blue. Afterward, platelets were analyzed by flow cytometry as described above.

### Preparation of Washed Neutrophils

Neutrophils were prepared from PB. Residual red blood cells were removed by means of magnetic beads using MACSxpress Whole Blood Neutrophil Isolation Kit (no.: 130–104–434) and MACSxpress Erythrocyte Depletion Kit (no.: 130–098–196) from Miltenyi Biotec B.V. & Co. KG (Bergisch Gladbach, Germany) according to the manufacturer's instructions.

### Platelet–Neutrophil Coincubation and Immunofluorescence Staining

Immunofluorescence glass chambers from ibidi GmbH (Graefelfing, Germany) were coated with 300 µL of 10 µg/mL poly-L-lysine in PBS for 30 minutes at 37°C. After washing of unbound poly-L-lysine, 300 µL of mixture containing 10 ^7^ platelets and 10 ^6^ neutrophils resuspended in Tyrode's salt solution were applied to each well, supplemented with 1 mM of CaCl _2_ and stimulated for 30 minutes at RT under continuous shaking according to Table 1 .

**Table 1:** Mixing protocol for platelet-neutrophil coincubation studies

TLR	Control	TLR activation	Inhibition of TLR activation
TLR2	7.5 µL PBS + 2.25 µL PBS	7.5 µL of 200 µg/mL isotype control for blocking anti-TLR2 antibody (5 µg/mL final concentration) + 2.25 µL of 2 mg/mL Pam3CSK4 (15 µg/mL final concentration)	7.5 µL of 200 µg/mL blocking anti-TLR2 antibody (5 µg/mL final concentration) + 2.25 µL of 2 mg/mL Pam3CSK4 (15 µg/mL final concentration)
TLR4	7.5 µL PBS + 2.25 µL PBS	7.5 µL of 200 µg/mL isotype control for blocking anti-TLR4 antibody (5 µg/mL final concentration) + 2.25 µL of 2 mg/mL LPS (15 µg/mL final concentration)	7.5 µL of 200 µg/mL blocking anti-TLR4 antibody (5 µg/mL final concentration) + 2.25 µL of 2 mg/mL LPS (15 µg/mL final concentration)

Abbreviations: LPS, lipopolysaccharides; TLR, toll-like receptor.

Note: Reagents as depicted are added to 300 µL of platelet–neutrophil mixture in each well.

After that, shaking was turned off and the coincubation was continued for the next 30 minutes to permit cell adhesion to the glass chamber. Hereafter, unattached cells were washed off with PBS and adherent platelets and neutrophils were fixed with 300 µL of cold 4% PFA in PBS for 15 minutes on ice. After fixation, adherent cells were washed with PBS, permeabilized for 10 minutes at RT with 300 µL of 0.2% Triton X-100 in PBS and blocked for 30 minutes at RT with 200 µL of PBS/GS/Triton X-100 (10% GS and 0.1% Triton X-100 in PBS).

Subsequently, slides were stained with 25 µg/mL mouse monoclonal antihuman CD41a and with 10 µg/mL of FITC-conjugated mouse monoclonal antihuman CD11b antibody diluted in 10% GS and 0.1% Triton X-100 in PBS overnight at 4°C. The next day, slides were washed five times with PBS and incubated with 0.4 mg/mL of the secondary TRITC-conjugated goat antimouse antibody for 30 minutes at RT. After mounting with ProLong Diamond Antifade Mountant and curing for 24 hours at RT, the slides were stored at 4°C until immunofluorescence acquisition. Mounted samples were imaged on an inverted Nikon Eclipse Ti2 microscope (Nikon GmbH, Duesseldorf, Germany) using a CFI Plan Fluor DLL 100X Oil objective and 1.5× zoom (14-bit digitalization). The analysis was performed at least in three fields per sample, assuring the same area. In each field, the neutrophils were identified and the number of platelets with direct contact to neutrophils were counted.

### Statistical Analysis

**Fig. 1:**
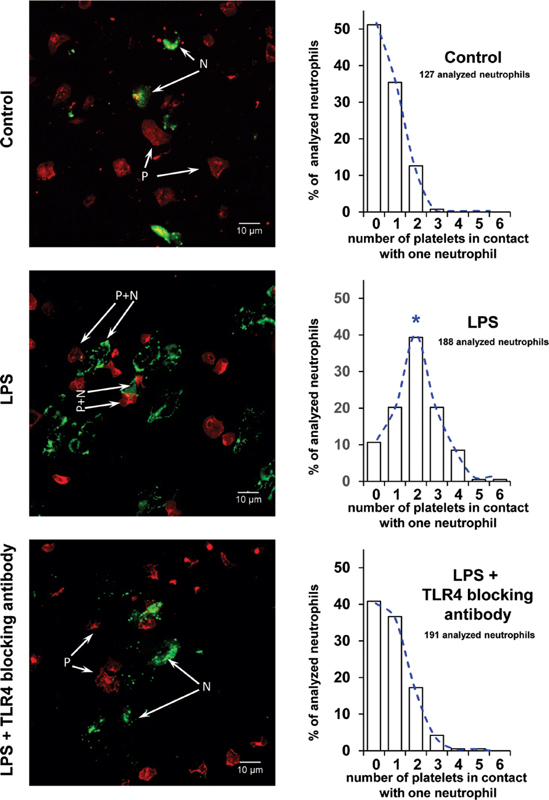
LPS increases the frequency of platelet–neutrophil contacts of adhered neutrophils. Left side: the representative images show adherent neutrophils and platelets on poly-L-lysine-coated slides after incubation with neutrophil-platelet mixtures, stimulated with LPS (“LPS”), stimulated with LPS in the presence of a TLR4 blocking antibody (“LPS + TLR4 blocking antibody”) or unstimulated (“Control”). Adherent platelets were stained with an anti-CD41a antibody and a TRITC-conjugated secondary goat anti-mouse antibody (red). Adherent neutrophils were stained with a FITC-conjugated anti-CD11b antibody (green). The slides were visualized with an inverted Nikon Eclipse Ti2 microscope using a 100× oil immersion objective and 1.5x zoom (14-bit digitalization). Right side: the quantification of neutrophil-platelet complex formation is shown. In total (as the sum of the four experiments), 127 neutrophils were identified on the slides for “control,” 188 neutrophils for “LPS” and 191 neutrophils for “LPS + TLR4 blocking antibody.” For each neutrophil, the number of platelets being in close contact to the neutrophils were assessed. The column diagrams show the distribution pattern of all counted neutrophils per setup according to the number of surrounding platelets (neutrophils in contact with 0 platelets, neutrophils in contact with 1 platelet, neutrophils in contact with 2 platelets, etc.). The distribution is given in percentage of the total number of analyzed neutrophils for “Control,” for “LPS” or for “LPS + TLR4 blocking antibody”; *n* = 4; *: *p* < 0.05; compared with “Control” and to “LPS + TLR4 blocking antibody.” LPS, lipopolysaccharides; TLR, toll-like receptor.

Descriptive data were calculated with GraphPad PRISM 7 (GraphPad Software, San Diego, California, United States). Data distribution analysis was performed using the Shapiro–Wilk test. Differences of variances between groups were analyzed by one-way analysis of variance (ANOVA) followed by post hoc Tukey–Kramer test for normally distributed data or by paired Student's *t* -test as appropriate. In the case of nonparametric distribution, the Kruskal–Wallis rank based test was used, followed by Dunn's multiple comparisons test. The comparison of distribution patterns in Fig. 1 was performed with the Kolmogorov–Smirnov test and rank analysis with the two-tailed Mann–Whitney test. *p* < 0.05 was considered statistically significant.

## Results

### Toll-Like Receptor-2 Stimulation Increases Toll-Like Receptor-2 and -4 Expression rather than Toll-Like Receptor-4 Stimulation

**Fig. 2:**
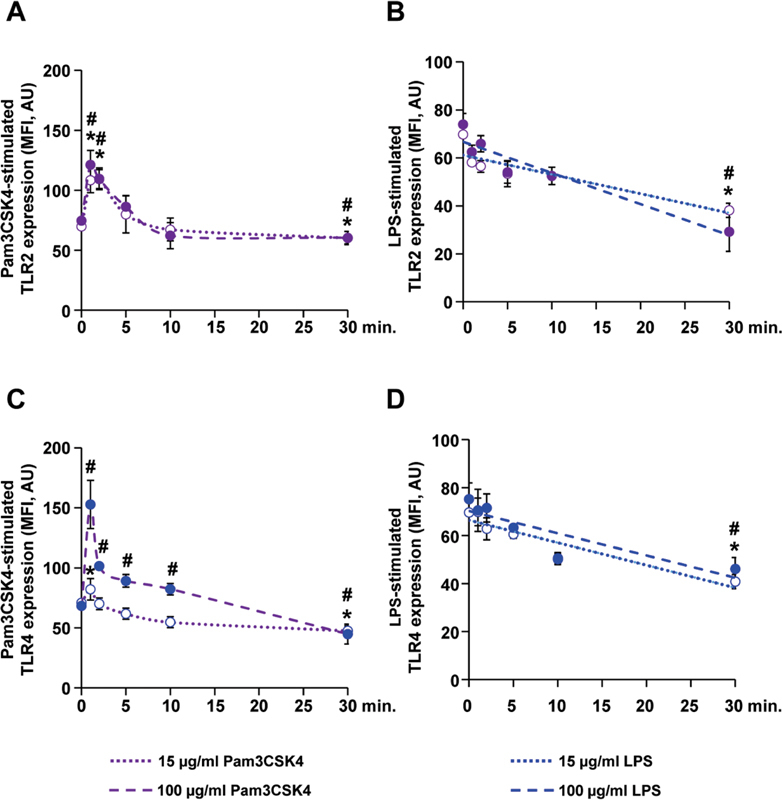
Pam3CSK4 induces a transient increase of TLR2 and TLR4 expression. TLR2 (purple circles) and TLR4 (blue circles) expression was measured in WP by flow cytometry after stimulation with 15 or 100 µg/mL of Pam3CSK4 (purple dotted line), **(A and C)** and 15 or 100 µg/mL of LPS (blue dotted line) **(B and D)** . Data are presented as mean MFI ± SEM; *n* = 3; *: *p* < 0.05; compared with control (0 minute), for 15 µg/mL of TLR ligands; # *p* < 0.05; compared with control (0 minute), for 100 µg/mL of TLR ligands. LPS, lipopolysaccharides; SEM, standard error of mean; TLR, toll-like receptor; WP, washed platelets.

Initially, the influence of TLR2 and TLR4 stimulation on time-dependent surface expression of both receptors in WP was analyzed ( Fig. 2 ) using the ligands Pam3CSK4 and LPS in the concentration of 15 µg/mL, and for comparison, in an additional higher concentration of 100 µg/mL. Also, 15 µg/mL Pam3CSK4 provoked an immediate increase of TLR2 expression from 69.8 ± 0.7 to 108.3 ± 10.4 MFI, thereby reaching peak values after 1 minute and returning to baseline within 10 minutes, followed by a slow decline to 60.1 ± 5.5 MFI ( Fig. 2A ) at 30 minutes. Further, 100 µg/mL Pam3CSK4 resulted in a similar course of TLR2 expression but with a slightly higher peak value of 121.1 ± 12.2 MFI at 1 minute after stimulation.

In contrast to Pam3CSK4, the TLR4 agonist LPS did not stimulate TLR2 expression under both agonist concentrations. During the incubation period of 30 minutes, a slow decline of TLR2 expression was observable, from 69.8 ± 0.8 to 38.2 ± 2.9 MFI with 15 µg/mL of LPS and to 29.2 ± 8.1 MFI with 100 µg/mL LPS ( Fig. 2B ).

The lower Pam3CSK4 concentration slightly increased TLR4 expression from 67.5 ± 2.5 MFI to 76.5 ± 1.6 ( Fig. 2C ) at 1 minute. Also, 100 µg/mL Pam3CSK4 induced TLR4 expression more intensively, with 152.9 ± 20.1 MFI. Similar to TLR2 expression, values of TLR4 expression decreased over 30 minutes to 47.5 ± 4.2 MFI after stimulation with Pam3CSK4. On 15 µg/mL LPS, a continuous decrease of TLR4 expression was induced from 69.6 ± 1.4 to 40.8 ± 3.0 MFI ( Fig. 2D ). Comparable values were observed with 100 µg/mL LPS.

### Toll-Like Receptor-2 Stimulation Induces the Expression of Platelet Activation Markers More Potently than Toll-Like Receptor-4 Stimulation

**Fig. 3:**
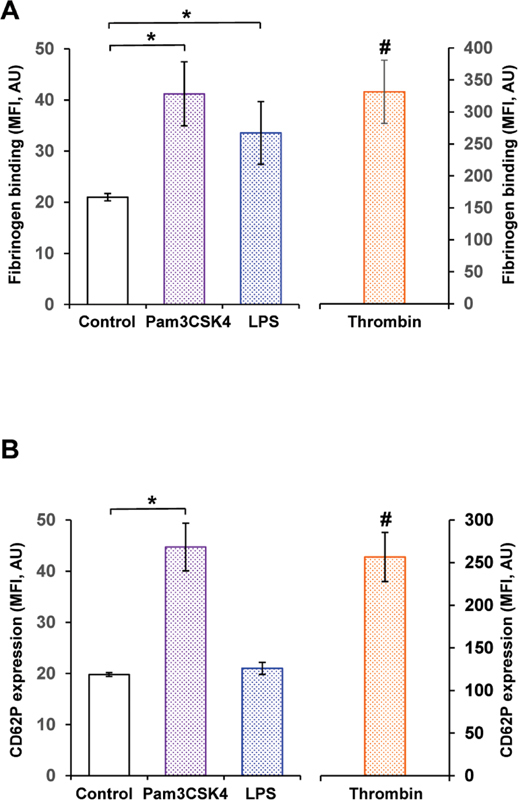
TLR2 stimulation results in weak platelet activation. Fibrinogen binding **(A)** and CD62P expression **(B)** were measured in WP after stimulation with 15 µg/mL of Pam3CSK4 (purple column), 15 µg/mL of LPS (blue column) or 0.5 U/mL thrombin (orange column) for 5 minutes. Data are presented as mean MFI ± SEM; *n* = 7; * *p* < 0.05; compared with control. # < 0.05; compared with control, Pam3CSK4- or LPS-stimulated samples. LPS, lipopolysaccharides; SEM, standard error of mean; TLR, toll-like receptor; WP, washed platelets.

The expression of platelet activation markers was determined by flow cytometry on TLR2 and TLR4 stimulation. Also, 15 µg/mL of Pam3CSK4 increased bound fluorophore-conjugated fibrinogen from 21.0 ± 0.7 to 41.2 ± 6.3 MFI ( Fig. 3A ). Further, 15 µg/mL of LPS also supported fibrinogen binding, but with a smaller increment, reaching 33.6 ± 6.1 MFI ( Fig. 3A ).

CD62P expression increased from 19.8 ± 0.4 to 44.7 ± 4.7 MFI on stimulation with 15 µg/mL of Pam3CSK4, whereas LPS was not able to change CD62P expression. Again, 0.5 U/mL thrombin stimulated fibrinogen binding to 331.5 ± 49.4 MFI ( Fig. 3A ) and CD62P expression to 256.7 ± 28.9 MFI ( Fig. 3B ).

### Toll-Like Receptor-2 Stimulation has a Higher Capacity to Induce Intracellular Reactive Oxygen Species Production than Toll-Like Receptor-4 Stimulation

**Fig. 4:**
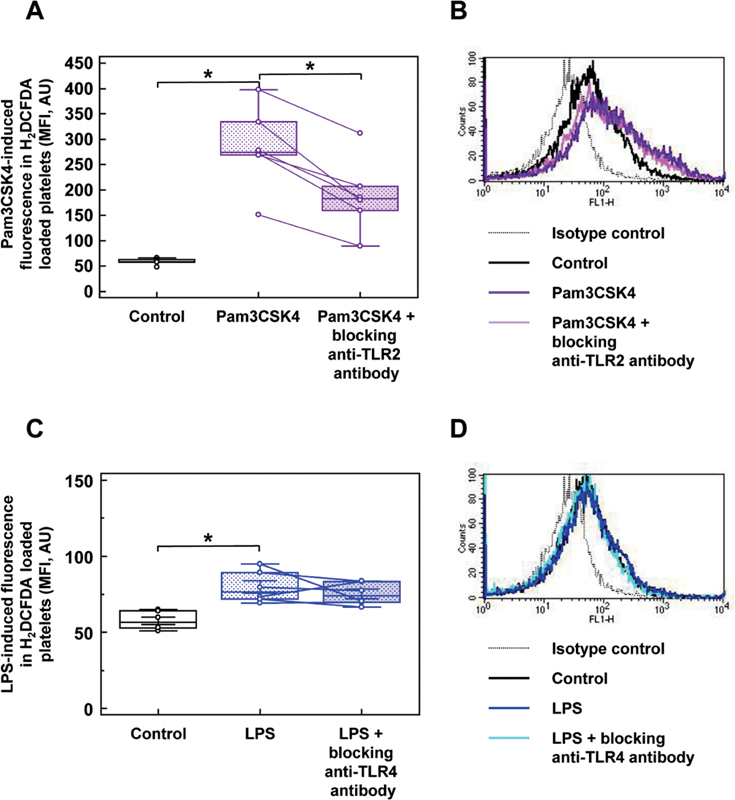
TLR2 and TLR4 activation evoke intracellular ROS production. The intracellular ROS production was measured in WP after stimulation with 15 µg/mL of Pam3CSK4 **(A and B)** and 15 µg/mL of LPS **(C and D)** . The data presented as box-and-whisker plots **(A and C)** demonstrate the changes of the single MFI values after TLR2 or TLR4 stimulation in the presence and absence of blocking antibodies. Data are presented as mean MFI ± SEM; *n* = 6; * *p* < 0.05; compared with control. The representative histograms of flow cytometry illustrate the shifts of mean fluorescence peaks under corresponding stimulations **(B and D)** . Further methodological details are given in Supplementary Fig. S1 . LPS, lipopolysaccharides; SEM, standard error of mean; TLR, toll-like receptor; WP, washed platelets.

In WP, intracellular ROS production was analyzed as a potential effector system of TLR2 and TLR4 by flow cytometry using the fluorescent substrate H _2_ DCFDA ( Fig. 4 ). Also, 15 µg/mL of Pam3CSK increased fluorescence of H _2_ DCFDA-loaded platelets from 56.3 ± 2.3 to 259.5 ± 36.6 MFI, whereas blocking of platelet TLR2 with a specific antibody resulted in the partial inhibition of Pam3CSK4-induced ROS-dependent fluorescence to 178.8 ± 26.6 MFI ( Fig. 4A ). Original histograms of flow cytometry show the shift of the MFI peak to the right after TLR2 stimulation and its shift to the left using the blocking antibody ( Fig. 4B ). Representative scatter diagrams illustrate the distribution of fluorescent H2DCFDA-loaded platelets before and after TLR stimulation ( Supplementary Fig. S1 ).

LPS induced only a small increase of ROS production from 57.7 ± 2.2 to 79.8 ± 3.7 MFI ( Fig. 4C and D ), without a significant inhibitory effect of the blocking anti-TLR4 antibody reaching values of 75.3 ± 2.8 MFI.

### Intracellular Uptake of Fluorophore-Labeled Toll-Like Receptor Ligands

**Fig. 5:**
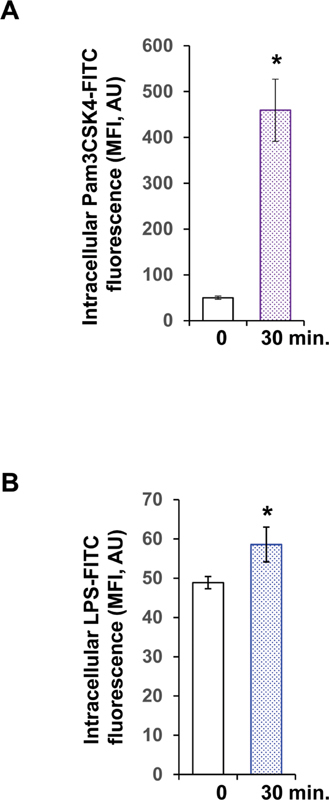
Intracellular uptake of fluorophore-labeled TLR ligands. The uptake of FITC-labeled Pam3CSK4 (A) or LPS (B) in WP was measured after 30 minute of stimulation. The detection of intracellular Alexa Fluor 488 fluorescence was performed by flow cytometry, after quenching of extracellular fluorescence with Trypan blue. Data are shown as mean MFI ± SEM; *n* = 6; * *p* < 0.05. LPS, lipopolysaccharides; SEM, standard error of mean; TLR, toll-like receptor; WP, washed platelets.

TLR trafficking into the cytoplasm was indirectly measured with FITC-labeled TLR ligands. After 30 minutes of stimulation with FITC-labeled Pam3CSK4, the intracellular fluorescence increased from 50.0 ± 3.5 to 459.3 ± 67.9 MFI ( Fig. 5A ). Platelet stimulation with FITC-labeled LPS resulted only in weak accumulation of fluorescence from 48.9 ± 1.6 to 58.6 ± 4.4 MFI after 30 minutes ( Fig. 5B ).

### Toll-Like Receptor-2 Mediates Fibrinogen Uptake in Platelets more Intensively than Toll-Like Receptor-4

**Fig. 6:**
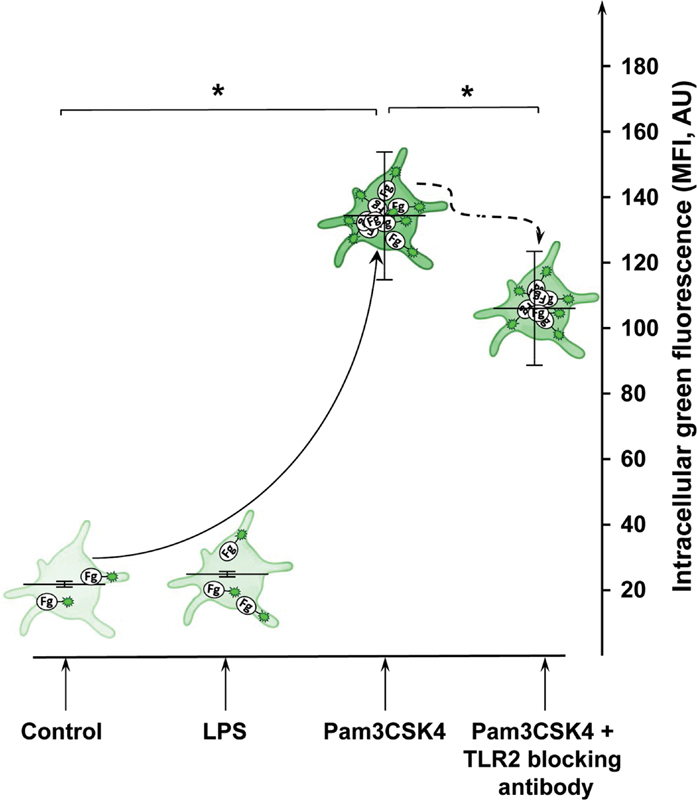
TLR2 stimulation activates fibrinogen uptake in platelets. The schematic illustration shows changes of intracellular green fluorescence in platelets stimulated with 15 µg/mL of Pam3CSK4 or 15 µg/mL of LPS in the presence of Alexa Fluor 488-conjugated fibrinogen (green). Data are presented as mean MFI ± SEM; *n* = 12; *: *p* < 0.05; compared with control. LPS, lipopolysaccharides; SEM, standard error of mean; TLR, toll-like receptor.

The ability of platelets to exert a TLR2- or TLR4-induced uptake of extracellular substances was investigated by flow cytometry using fluorophore-conjugated fibrinogen ( Fig. 6 ). After stimulation of WP with Pam3CSK4 for 1 hour, intracellular green fluorescence increased from 20.3 ± 0.9 to 137.3 ± 19.5 MFI. Addition of the blocking anti-TLR2 antibody partially inhibited the uptake to 106.3 ± 17.4 MFI. LPS did not trigger the uptake of fibrinogen.

### Toll-Like Receptor-4 but Not Toll-Like Receptor-2 Promotes Platelet–Neutrophil Interactions

To elucidate TLR2- and TLR4-dependent interactions with immune cells, coincubation experiments were performed ( Fig. 1 ). In the presence of Pam3CSK4 or LPS, WP were added to suspensions of washed neutrophils in vitro and analyzed by fluorescent staining.

Visually, the incubation with LPS, but not with Pam3CSK4, resulted in a remarkable increase of platelet–neutrophil contacts ( Fig. 1 , left side). The distribution pattern of neutrophils being in contact with a certain number of platelets shifted from “0 or 1 contacting platelets” per neutrophil to “2 or more contacting platelets” per neutrophil under LPS ( Fig. 1 , right side).

In average, one neutrophil had direct contact to 1.00 ± 0.02 platelets in the absence of LPS, rising up to 2.54 ± 0.35 platelets per neutrophil in the presence of LPS. The addition of a blocking anti-TLR4 antibody inhibited this effect, reducing the number of platelet–neutrophil contacts to 0.91 ± 0.24 platelets per neutrophil.

Under LPS stimulation, neutrophils showed an emphasized CD11b staining located at the cell membrane, indicating neutrophil activation. In contrast, Pam3CSK4 did not change the distribution of adherent platelets and neutrophils compared with unstimulated cells on control slides.

Since CD40L may play an important role in interactions of platelets and neutrophils, platelet CD40L expression was determined after stimulation of TLR2 and TLR4 ( Supplementary Fig. S2 ). In WP, Pam3CSK4 induced an increase from 23.9 ± 1.0 to 131.8 ± 5.0 MFI, whereas LPS and thrombin did not change the levels. In PRP, CD40L expression remained unaffected by Pam3CSK4, LPS, or TRAP-6.

## Discussion

Platelets are essential players in thrombosis and hemostasis. Moreover, being equipped with chemokines or immune receptors, like TLR2 or TLR4, they are considered to be a part of the innate immunity system. In this study, we analyzed nonhemostatic functions of platelets mediated by TLR2 and TLR4, potentially contributing to immunological mechanisms.

For stimulation of TLR2 or TLR4, Pam3CSK4 or LPS were used as specific ligands. Concentrations were chosen in accordance to our previous studies to achieve submaximal hemostatic platelet activation and to avoid unspecific overstimulation. 8 9 The analysis of TLR expression levels were additionally performed with a higher concentration to recognize dose-dependent stimulatory effects.

First, we addressed the maintenance of TLR2- and TLR4-receptor expression after ligand binding. Similar to its ability to induce aggregation and cytoskeletal rearrangements, 15 µg/mL of Pam3CSK4 increased TLR2 expression more than two-fold and TLR4 expression by approximately 15% on the platelet surface membrane. This effect was transient for a short period of 1 minute, followed by reversal to baseline levels ( Fig. 2A and C ). Under the higher 100 µg/mL of Pam3CSK4 concentration, an additional stimulatory effect was observable for TLR4 expression but not for TLR2 expression. Obviously, platelets contain a storage pool of TLRs, with a higher dose-dependent capacity for TLR4, available for rapid redistribution and to emphasize responses upon TLR2 activation. Such a mechanism has also been proposed for the recruitment of platelet purinergic receptors P2Y1 and P2Y12 to the platelet surface via mobilization of additional receptors from the α-granule membrane and from the open canalicular system-associated pool. 17 18 19

TLR4-mediated activation was not associated with relevant changes in TLR2 or TLR4 expression directly after stimulation with the two used LPS concentrations. One reason for the continuous decrease in expression could be caused by the deterioration of washed platelets during prolonged storage 20 associated with TLR trafficking into the cytoplasm. The indirect measurement of intracellular TLR uptake was performed with fluorophore-labeled ligands. FITC-labeled Pam3CSK4 and FITC-labeled LPS both accumulated in platelets after 30 minutes of stimulation, indicating internalization of TLR together with their ligands.

For TLR2, the amount of intracellular Pam3CSK4 strongly increased, suggesting an intensive trafficking of TLR2 to the membrane, and then, back to the cytoplasm together with the bound ligand, potentially for destruction by ROS. In contrast, FITC-labeled LPS only weakly accumulated in platelets pointing to low trafficking of TLR4. Probably, TLR4 is shed from the surface on activation, as similarly described for endothelial cells. 21

CD62P is an important marker of platelet activation and, in addition, a major binding molecule for platelet–leukocyte interactions. 10 22 LPS was not able to affect CD62P expression, whereas Pam3CSK4 elevated the levels more than two-fold, similar to the results shown for TLR expression. At the same time, fibrinogen binding as a prerequisite of aggregation 23 was induced by Pam3CSK4 but only to a minor degree for LPS, not sufficient for direct initiation of aggregation. 9

CD40L expression, another relevant molecule for platelet–leukocyte interaction, 24 was increased on stimulation with Pam3CSK4 but not with LPS, confirming the results of minor sCD40L release on LPS incubation of WP. 9 Considering the lower capacity of TLR4 to initiate platelet activation, aggregation and intracellular activating signal pathways, together with its ability to stimulate PP2A and to promote ristocetin-induced agglutination, platelet adhesion 8 or in vivo thrombus formation, 25 it appears that TLR4 preferably regulates the conformational state of platelet adhesion receptor GPIb.

A powerful tool of leukocytes to neutralize microbiotic antigens is phagocytosis of particles followed by intracellular destruction. For platelets, an uptake of serotonin, cytokines, or other substances from the surrounding environment has been described. 26 27 28 29 Furthermore, platelets possess a machinery to produce intracellular ROS. 14 15 In this regard, we could show for the first time that both investigated platelet TLR are involved in mechanisms of intracellular ROS generation. Stimulation of TLR2 resulted in a rapid and five-fold increase of intracellular ROS, whereas stimulation of TLR4 resulted only in a smaller increase of approximately 50%. In addition, uptake-experiments revealed that platelets are able to incorporate fluorescent-conjugated fibrinogen with a higher intensity for Pam3CSK4 than for LPS stimulation. In this context, the observed time-delayed progressive decrease of TLR2 and TLR4 expression after stimulation with Pam3CSK4 and LPS may also indicate ongoing TLR2/4 internalization into endosomes, together with bound ligands to neutralize exogenous pathogenic substances.

Similar to ROS analysis, the blocking antibody only partially suppressed fibrinogen uptake which may be caused by different affinities of ligands and blocking antibodies allowing a residual stimulation of the receptors. The inhibitory effect of the blocking antibody may be directed to the inhibition of transmembrane signaling rather than intracellular signaling like the NFκB pathway. 6 30 31 The fact that TLR2 and TLR4 form complexes with TLR1 or TLR6 and CD14 6 could be another explanation for incomplete inhibition. Fibrinogen may be internalized together with αIIbßIII into platelets after induced coactivation via released ADP on TLR2 stimulation. 32

Notably, incubation with LPS demonstrated only marginal effects on CD62P expression, ROS production, or fibrinogen uptake. However, in general, LPS stimulation is able to induce platelet responses, for example, leading to chemokine release or supported thrombin-induced threshold aggregation in WP. 9 In addition, the presence of CD14 or MD2—as suggested obligatory components of the TLR4 complex—has been confirmed on the membrane surface of WP. 33 In this study, we can show that LPS substantially induced the formation of platelet–neutrophil complexes. Since LPS had the capacity to increase platelet agglutination after costimulation with ristocetin, 8 it may be concluded that TLR4-mediated signaling modulates the reactivity state of platelet GPIb. 8 34 35 The interaction of platelet GPIb and macrophage-1 antigen (MAC-1) on the surface of immune cells plays an essential role in the mechanisms that promote the recruitment of immune cells to the site of injury and inflammation. 34 36

## Limitations

As a limitation of the study, it should be mentioned that we used the lowest concentration of TLR ligands, eliciting hemostatic responses. Higher concentrations may lead to more enhanced immune-related effects, for example, for LPS-related effects. However, the analytical procedures comprised several functional systems, and obtained results were consistent with previously published data, highlighting differences in TLR2- and TLR4-mediated responses. In this context, it would be of interest to investigate intracellular pathways associated with TLR2- or TLR4-dependent interactions of platelets with leukocytes in addition to further dose-response experiments. Moreover, it should be considered that platelets interact in vivo not only with leukocytes but also with red blood cells or endothelial cells and react to changes in plasma composition. The intention of this in vitro study was to analyze platelet-specific responses, preferably using washed platelets for experimentation. The influence of other cell types upon exposition of platelets to TLR ligands will be addressed comprehensively in future studies, for example, by using flow cytometry and live cell imaging under flow conditions.

In adhesion studies, fluorescent staining of CD11b revealed a predominant membrane localization in neutrophils. Therefore, it cannot be ruled out that residual LPS exerted activating effects on neutrophils. However, in case of bacterial infections both kinds of cells would be exposed to LPS simultaneously. These observations are in accordance with previous reports showing that 300 ng/mL LPS significantly increases intercellular adhesion molecule (ICAM) expression on murine neutrophils, 37 whereas 10 ng/mL LPS induces CD11b expression and decreases CD62-L expression 38 in human neutrophils. Furthermore, former studies demonstrated that LPS (1 ng/mL) enhanced MAC-1 expression in neutrophils, 39 allowing the suggestion that LPS regulates both the platelet GPIb reactivity and the neutrophil MAC-1 expression at the same time.

## Conclusion

**Fig. 7:**
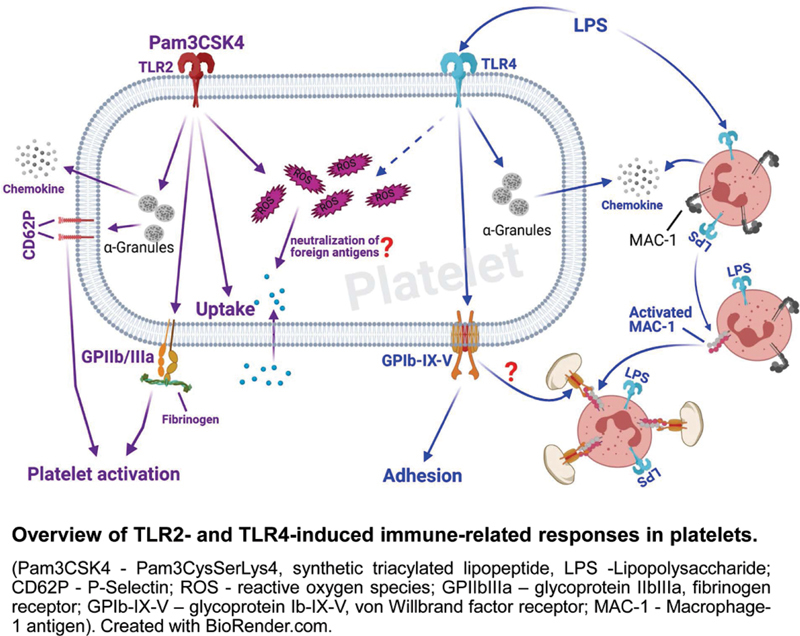
Visual summery. TLR, toll-like receptor.

In conclusion, this study has revealed novel immune-related responses of platelets on TLR2 and TLR4 stimulation using bacterial ligands. The activation of TLR2, particularly results in ROS production and uptake of molecules like fibrinogen. In contrast, activation of TLR4 mediates the formation of platelet–neutrophil complexes. In this way, different responses of platelets may be initiated upon TLR activation dependent on binding of specific ligands ( Fig. 7 ).

## Notes

**What Is Known on This Topic?**

- Platelets bear toll-like receptors 2 and 4 (TLR 2 and TLR4) on their membrane surface.
- Stimulation of TLR2 and TLR4 with agonists leads to platelet activation via different signaling pathways.

**What Does This Paper Add?**

- TLR2 and TLR4 show different nonhemostatic, immune-related responses on activation, potentially important for bacterial defense.
- TLR2 stimulation particularly results in the production of reactive oxygen species and in the uptake of molecules like fibrinogen.
- TLR4, but not TLR2, triggers the formation of platelet–neutrophil complexes.
