# Supplementary material for: Platelet Toll-Like-Receptor-2 and -4 Mediate Different Immune-Related Responses to Bacterial Ligands
Source: TH Open. 2022 Jul 11;6(3):e156–67. doi: 10.1055/a-1827-7365 (PMC9273317; doi:10.1055/a-1827-7365)
Supplement: Supplementary file 1 — Supplementary Material [file 10-1055-a-1827-7365-s220006.pdf]

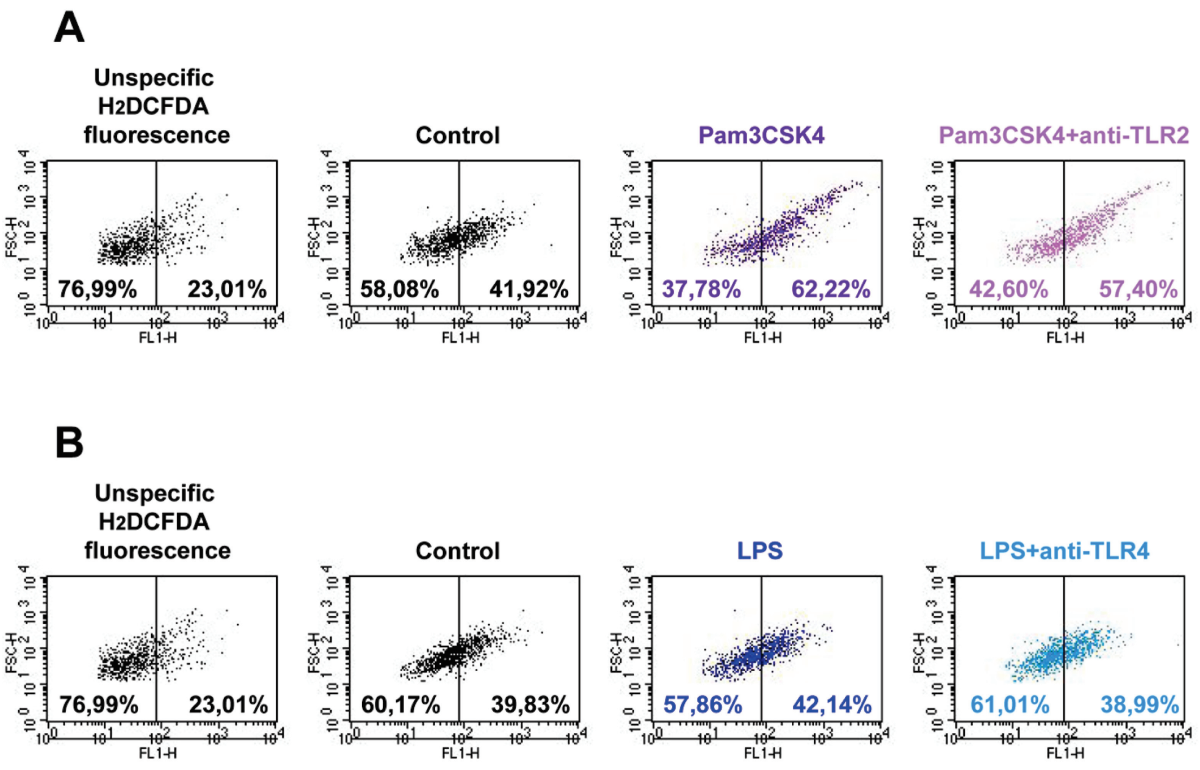

Supplementary Fig. S1 Flow cytometric analysis of ROS production. Representative scatter diagrams illustrate the distribution of fluorescent H2DCFDA-loaded platelets after stimulation with 15 µg/mL of Pam3CSK4 **(A)** or 15 µg/mL of LPS **(B)**. For comparison, unspecific fluorescence of the PFA fixed platelets coincubated with H2DCFDA, control samples (incubated without addition of Pam3CSK4 or LPS), and samples with blocking antibodies (specific rat polyclonal blocking antibodies for human TLR2 or TLR4) are shown. $n = 6$. LPS, lipopolysaccharides; ROS, reactive oxygen species; TLR, toll-like receptor.

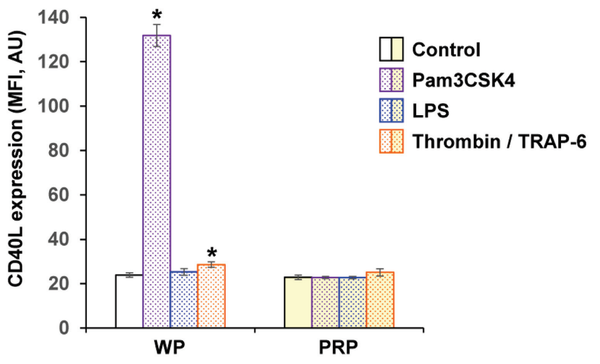

Supplementary Fig. S2 TLR2 and TRL4-mediated effects on CD40L expression. CD40L expression was measured by flow cytometry in WP and PRP after stimulation with 15 µg/mL of Pam3CSK4, 15 µg/mL of LPS, 0.5 U/mL of thrombin (in WP), or 10 µM TRAP-6 (in PRP) for 5 minutes. Data are presented as mean MFI ± SEM; $n = 8$; *: $p < 0.05$; compared with control. LPS, lipopolysaccharides; PRP, platelet rich plasma; SEM, standard error of mean; TLR, toll-like receptor; WP, washed platelets.

 © 2022. The Author(s).
